# Scientific basis and applicability of the ISO 2631–1 health guidance caution zone

**DOI:** 10.1007/s00420-026-02236-0

**Published:** 2026-09-06

**Authors:** Marco Tarabini, Genevieve Williams, Suzanne Smith, Setsuo Maeda, Massimo Bovenzi

**Affiliations:** 1 Department of Mechanical Engineering, Politecnico Di Milano, Milan, Italy; 2 Public Health and Sport Sciences, University of Exeter, Exeter, UK; 3 Consultant/Research Engineer, Dayton, OH USA; 4 Department of Engineering, Nottingham Trent University, Nottingham, UK; 5 Clinical Unit of Occupational Medicine, Department of Medical Sciences, University of Trieste, Trieste, Italy

**Keywords:** Whole-body vibration, Health guidance caution zone, ISO 2631–1, Occupational musculoskeletal disorders, Vibration exposure assessment

## Abstract

**Purpose:**

This work evaluates the scientific foundation and practical applicability of the Health Guidance Caution Zone (HGCZ) defined in the ISO 2631–1 standard for assessing health risks from whole-body vibration. The primary objective is to investigate the HGCZ’s derivation process, its underlying data sources, and its effectiveness in predicting adverse health outcomes across various occupational environments.

**Methods:**

This study examines the relationship between vibration metrics—such as frequency-weighted acceleration and vibration dose value—and reported musculoskeletal, neurological, and vascular outcomes, while also considering the role of individual confounding factors, including posture, age, and body mass index, in shaping exposure–response relationships.

**Results:**

HGCZ boundaries were identified as pragmatic expert compromises rather than being derived from quantitative dose–response relationships. While the zone is a reasonable predictor for low back pain in seated operators, it lacks empirical support for neck, shoulder, or vascular outcomes. Analysis of aviation data showed that most flight scenarios reached caution thresholds significantly faster than traditional land-based driving, while individual factors such as posture and body mass index were found to modify actual health outcomes.

**Conclusion:**

We suggest the evolution of the HGCZ toward a probabilistic framework, where fixed thresholds are complemented by models linking exposure magnitude and duration to the likelihood of specific health outcomes (not only low back pain). Supported by biomechanical modelling and segment-specific frequency weightings, this approach would enable a more accurate assessment of WBV risk, extending rather than replacing the current framework.

## Introduction

Standardized methods for evaluating human exposure to whole-body vibration (WBV) are essential for assessing potential health risks across different field environments. In occupational health, standards like the ISO 2631–1:1997/Amd 1:2010, ‘‘Mechanical vibration and shock—Evaluation of human exposure to whole-body vibration’’ and BS 6841”Guide to measurement and evaluation of human exposure to whole-body mechanical vibration and repeated shock” are widely used to quantify exposure for operators of vehicles and machinery, informing risk management strategies and providing the basis for the definition of regulatory limits (Directive—2002*/44—EN—EUR-Lex*, n.d.). The potential adverse health effects associated with long-term WBV exposure, particularly musculoskeletal disorders (MSD) such as low back pain (LBP) and degenerative spinal changes, are well-documented in epidemiological studies of adult working populations (Bovenzi 1996, 2010).

This work focuses on the Health Guidance Caution Zone (HGCZ) as presented in the ISO 2631–1:1997 standard and its 2010 amendment, and in the version currently under revision in an ad hoc ISO Committee. This analysis summarizes the available information regarding the derivation process of the HGCZ, its underlying data sources (or lack thereof), its intended interpretation, and the known critiques and limitations documented in scientific literature and related standards documents.

The literature was not analysed through a formal systematic review protocol; instead, a targeted and iterative selection process was adopted. Relevant studies were identified based on their contribution to key themes, including epidemiological evidence on WBV and health outcomes, methodological approaches to exposure assessment, and investigations of confounding and modifying factors. Priority was given to influential review papers and studies frequently cited in the WBV field, as well as works addressing emerging contexts such as aviation and marine environments. Our analysis focuses on synthesizing and critically interpreting the evidence rather than providing an exhaustive survey, with the objective of highlighting conceptual limitations of the HGCZ and supporting the development of more advanced, data-driven risk assessment frameworks.

### The health guidance caution zone

The HGCZ is defined in Annex B of the ISO 2631–1:1997 standard. Annex B, informative in nature, provides guidance rather than mandatory requirements. The stated purpose of the HGCZ is to offer a framework for evaluating the potential health risks associated with exposure to WBV. Its declared context of application is occupational health, particularly for assessing risks to seated workers exposed to multi-axis translational vibration generated by vehicles or industrial machineries. The guidance aims to help determine when vibration exposure levels might warrant concern and potentially require mitigation measures.

ISO 2631–1:1997 specified two main quantitative methods for assessing vibration effects. Both methods are based on measuring the acceleration on the surface transmitting vibration to the body:

- Frequency-Weighted R.M.S. Acceleration, which is presented as the basic evaluation method. The measured accelerations in the three orthogonal axes $a_{x} \left( t \right)$, $a_{y} \left( t \right)$, and $a_{z} \left( t \right)$ are frequency-weighted with weightings $W_{d}$ and $W_{k}$ for horizontal and vertical axes, respectively, to reflect the human body's varying sensitivity to different vibration frequencies regarding potential health effects, obtaining $a_{wx} \left( t \right)$, $a_{wy} \left( t \right)$ and $a_{wz} \left( t \right)$. Time histories are then integrated to derive, for each axis, the weighted r.m.s. values:
  $$ a_{wx} = \sqrt {\frac{1}{T}\mathop \smallint \limits_{0}^{T} \left[ {a_{wx} \left( t \right)} \right]^{2} dt} $$
  $$ a_{wy} = \sqrt {\frac{1}{T}\mathop \smallint \limits_{0}^{T} \left[ {a_{wz} \left( t \right)} \right]^{2} dt} $$
  $$ a_{wz} = \sqrt {\frac{1}{T}\mathop \smallint \limits_{0}^{T} \left[ {a_{wz} \left( t \right)} \right]^{2} dt} $$
- Multiplying factors $k_{x}$, $k_{y}$, and $k_{z}$ are applied to derive the vibration total value $a_{w}$: $k_{x}$ and $k_{y}$ are equal to 1.4, while $k_{z}$ is 1.
  $$ a_{w} = \sqrt {\left( {k_{x} a_{wx} } \right)^{2} + \left( {k_{y} a_{wy} } \right)^{2} + \left( {k_{z} a_{wz} } \right)^{2} } $$
  In case the vibration along a single axis is dominant, the worst axis criterion shall be used instead of the vibration total value.
- Vibration Dose Value (VDV), which is recommended when the vibration contains occasional but significant shocks. VDV is calculated based on the fourth power of the frequency-weighted r.m.s. accelerations integrated over the measurement duration. Since the VDV is dependent on time, the measured value must be modified to reflect the occupant’s exposure relative to the exposure duration (if not equal to the measurement duration).
  For each axis $l \in \left\{ {x,y,z} \right\}$, the VDV over an exposure duration *T* is computed as.
  $VDV_{l} = \left( {\mathop \smallint \limits_{0}^{T} a_{wl}^{4} \left( t \right)\,dt} \right)^{1/4}$ with units of ${\mathrm{m}}/{\mathrm{s}}^{1.75}$. For multiple exposure periods *i*, partial VDV values ar combined as
  $$ VDV_{l} = \left( {\mathop \sum \limits_{i} VDV_{l,i}^{4} } \right)^{1/4} ,VDV_{l,i} = \left( {\mathop \smallint \limits_{0}^{{T_{i} }} a_{wl,i}^{4} \left( t \right)\,dt} \right)^{1/4} . $$

Axis multiplying factors are applied to each axis, yielding

$VDV_{l}^{*} = k_{l} \,VDV_{l} .$ The daily vibration exposure is finally taken as the maximum across the three orthogonal axes. Unlike RMS-based metrics, VDV is proportional to the fourth power of acceleration and is therefore more sensitive to transient shocks and peaks. For approximately stationary vibration, VDV scales with exposure duration as ${\mathrm{VDV}} \propto T^{1/4}$.

The existence of these two distinct methods within the same standard reflects the presence, in the scientific literature, of works suggesting that one method may be more conservative as compared to the other in predicting the potential hazard of vibration in different occupational settings. Studies (Bovenzi 2009) have shown that the choice between A(8) and VDV can lead to different risk classifications for the same exposure scenario.

**Fig. 1:**
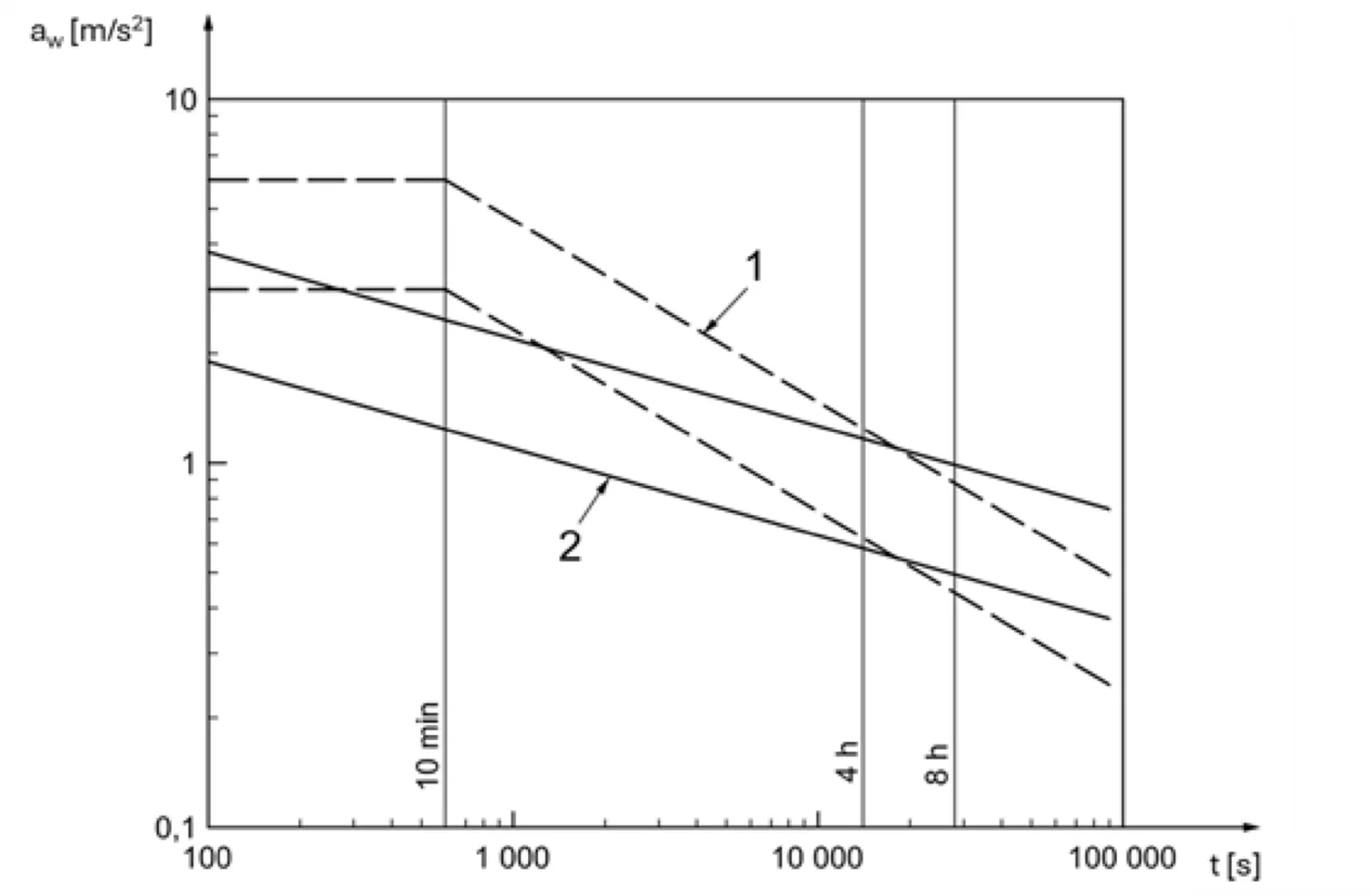
HGCZ reported in the amendment 2010 of ISO 2631. Dashed lines (1): Equation B.1 (Basic Evaluation Method). Solid lines (2): Equation B.2 (Fourth Power Method) (lines represent constant estimated VDV). X axis: daily exposure time [s], Y axis: weighted r.m.s. vibration

There is a HGCZ defined by specific numerical boundaries associated with each of the two quantitative methods described above. The two HGCZs are visually represented as regions on the plot of Fig. 1.

The interpretation provided within the standard and in the known literature suggests a probabilistic approach to risk:

- *Below the lower boundary line 1 or 2*: The standard indicates that adverse health effects ‘‘have not been clearly documented and/or objectively observed’’, implying a low likelihood of risk based on the evidence considered for the standard.
- *Above the upper boundary line 1 or 2*: The standard states that ‘‘health risks are likely’’, evidencing a consistent probability of adverse health effects, thus triggering requirements for immediate action to reduce exposure in regulatory contexts.
- *Between the upper and lower lines 1 or 2*: The standard advises that "caution with respect to potential health risks is indicated".

To the authors’ knowledge, the exposure thresholds were introduced as practical compromises rather than values derived from precise evidence. The 1997 version of the standard states that the HGCZ is based on continuous exposures with durations between 4 and 8 h, and that the application to shorter durations requires caution. The 2010 amendment defined the lower and upper boundaries for the dashed lines of 3 m/s^2^ r.m.s. and 6 m/s^2^ r.m.s., respectively, for exposures lasting 10 min or less. The lower and upper solid lines represent constant VDVs (based on the estimated VDVs or eVDVs) of 8.5 m/s^1.75^ and 17 m/s^1.75^, respectively.

These numerical values lend an appearance of quantitative precision to the HGCZ. However, the standard itself notes that health effects below the zone are “not clearly documented,” and broader critiques highlight the lack of a robust epidemiological basis. Therefore, these values should be interpreted with caution: they represent pragmatic demarcations established by the ISO TC108/SC4/WG13 committee, based on the information and expert consensus available prior to 1997 in specific occupational contexts. The use as thresholds derived from quantitative dose–response is therefore unreasonable (Griffin 2004).

### Scientific foundation and applicability of the HGCZ

A challenge in assessing the scientific foundation of the HGCZ is the standard's lack of explicit documentation linking the HGCZ figure and its boundaries to specific studies within its bibliography or detailing the methodology used for its derivation and extrapolation (Johanning and Turcot 2025).

### Origin and derivation of the HGCZ

The development and maintenance of the ISO 2631 series of standards falls under the responsibility of the International Organization for Standardization's Technical Committee ISO/TC108 (Mechanical vibration, shock and condition monitoring) and its Subcommittee SC 4 (Human exposure to mechanical vibration and shock). The first version of ISO 2631 was published in 1978 and first revised in 1985. A second review was registered in the technical committee work program in 1989 and was published in 1997.

BS 6841, published in 1987, introduced a VDV limit of 15 m/s^1.75^ and was developed to provide interim guidance in the UK while many ISO 2631 criteria were still under development.

## Conceptual foundations of the HGCZ

While the standard does assume that vibration responses are related to energy, as defined by Equations B.1 and B.2 in Annex B of ISO 2631–1, the technical rationale for the specific values used in the HGCZ is not explicitly detailed, neither in the standard nor in ISO technical documents.

Given the lack of explicit derivation in the ISO standard itself and the influence of preceding standards, the specific HGCZ boundaries were proposed and agreed upon internally by the expert committee, based on the available data and consensus at the time.

### Epidemiological evidence linking WBV to health outcomes

After the introduction of ISO 2631–1:1997, several studies used the standard to measure, evaluate and assess the effects of WBV on health, aiming to correlate the standard's metrics with specific pathologies. Health effects associated with WBV exposure have been reviewed by Krajnak (Krajnak 2018); WBV has been associated with an increased risk of lumbar spine MSD, neck and shoulder pain, vascular and neurological disease. The following sections summarize key studies that have linked specific health disorders to the vibration exposure metrics defined in ISO 2631–1. Additionally, a list of potential confounding factors is provided, which may inform future modifications to the HGCZ.

#### Lumbar spine disorders

The prevalence of LBP among professional drivers exposed to WBV has been investigated by Bovenzi et al. (Bovenzi et al. 2006). *A*(8) was in the range between 0.10 and 1.18 m/s^2^ rms and the 7-day and 12-month prevalence of LBP was greater in the driver groups than in the controls. In another epidemiological study performed on urban bus drivers (Bovenzi and Zadini 1992), low back symptoms occurred at WBV 8-h equivalent exposure levels of 0.4 m/s^2^ rms, i.e. at values in the vicinity of the lower HGCZ boundary of 0.43 m/s^2^ rms at 8 h, but values lower than the exposure action value of 0.5 m/s^2^ rms established by the EU Directive on mechanical vibration. Bovenzi and colleagues (Bovenzi et al. 2002) investigated the prevalence of LBP among port machinery operators exposed to WBV and postural load, comparing them to a control group. Their findings indicated a significantly higher prevalence of low back symptoms among the drivers of fork-lift trucks (average WBV total value or vector sum of 0.9 m/s^2^ rms) compared to either the control men (maintenance workers who performed manual activities at the same company and were not exposed to WBV) or the drivers of mobile cranes and overhead cranes (average WBV total values of 0.53 and 0.22 m/s^2^ rms, respectively). The study identified a potential dose–response relationship demonstrating that the prevalence of low back symptoms increased proportionally with the duration of exposure, equivalent vibration magnitude, and cumulative vibration exposure, thus evidencing the validity of ISO 2361–1 framework.

The relation between A(8) and VDV estimates and LBP in professional drivers has been also investigated in ref. (Bovenzi 2009). The incidence of 12-month LBP in a cohort of 537 drivers over a 2 year follow-up period evidenced that measures of vibration exposure derived from VDV and exposure duration were better predictors of LBP outcomes over time than measures of vibration exposure based on A(8).

Burström and colleagues (Burström et al. 2015) conducted a systematic review of the literature to evaluate the association between WBV exposure estimates and LBP. Their review strongly suggested that there is scientific evidence that exposure to higher vibration magnitudes increased the risk of LBP and sciatica, but there are insufficient data to determine levels at which risk is not increased.

Joseph and colleagues (Joseph et al. 2020) systematically reviewed the literature related with the prevalence of musculoskeletal disorders among professional drivers. Results evidenced heterogeneity in musculoskeletal problems across different body regions. The low back was the most frequently affected area, followed by the neck, shoulder, and upper back. The differential occurrence across anatomical segments raises pertinent questions regarding the suitability of applying a single HGCZ for risk assessment and prevention.

Since 2020, a number of systematic reviews related to the effects of WBV have been published, synthesizing the strengths and limitations of existing knowledge in the area. Pickard et al. (Pickard et al. 2022) concluded that lower back pain and upper back pain are the most common musculoskeletal disorders in occupational driving, particularly in truck, bus, and taxi drivers. The number of working years and working hours have also been associated with the prevalence of MSD amongst drivers. In heavy equipment and vehicle operators, Singh et al., (Singh et al. 2024) systematically reviewed up-to-date knowledge and also emphasised the reports of lower back pain as well as upper back pain.

The existing studies indicate a clear association between WBV exposure and LBP disorders and the general reasonableness of adopted metrics and thresholds indicated in the standard for vehicles drivers; the lower back is consistently identified as the most affected region.

#### Other musculoskeletal disorders

A cross-sectional study investigating the association between self-reported occupational WBV exposure and neck pain in a large general population sample found a significant association between reporting frequent occupational WBV exposure and reporting neck pain (Stjernbrandt et al. 2024). This association was statistically significant among men, suggesting that occupational WBV exposure may be a relevant risk factor for neck pain, but since WBV exposure was not quantified it is impossible to establish a dose–response relationship.

Another study investigated the neck and arm pain among professional forest machine drivers, examining its potential relationship with WBV exposure (Rehn et al. 2009). Using a survey and calculating various WBV exposure and dose measures, the study found a prevalence of neck pain of 34%, frequently combined with arm pain. However, no significant association between the WBV exposure measures and either the presence or the type of neck pain was found. While drivers with neck pain more often perceived shocks and jolts as uncomfortable, the objective WBV characteristics calculated did not appear to explain the occurrence or nature of neck pain in this population.

In a three-year follow-up study, the occurrence of neck and shoulder pain in terms of frequency, duration and intensity was investigated in a population of professional drivers (Stjernbrandt et al. 2024). Over the follow-up period, the cumulative incidences for neck and shoulder pain were 31.9% and 21.4%, respectively. After adjustment for potential confounders, a measure of cumulative WBV exposure (m^2^s^−4^ h) was significantly associated with neck-shoulder outcomes. However, adverse ergonomic risks factors (e.g. lifting loads, driving with trunk bent or twisted), poor psychosocial environment, and psychological distress were also related to neck and shoulder pain, suggesting a multifactorial origin of neck-shoulder outcomes in driving occupations.

Overall, the epidemiological evidence linking WBV exposure to neck and shoulder disorders is limited. Studies show mixed results and a strong influence of confounding ergonomic and psychosocial factors. While some associations have been reported, the lack of robust dose–response relationships and the variability in exposure characterization suggest that current WBV metrics may not be adequate predictors of neck and shoulder outcomes. This indicates the need for alternative or extended assessment approaches, potentially incorporating segment-specific metrics, posture-sensitive measurements, and additional descriptors of vibration exposure.

#### Neurological and vascular effects

Neurological and vascular effects associated with WBV are primarily observed in scenarios where vibration is transmitted through the feet rather than through a seated interface. In conventional ground-vehicles, seat cushions and suspensions attenuate higher-frequency vibration components, which are known to be more relevant for vascular and neurological responses (K. A. Goggins et al. 2019a). As a result, these types of effects more common in standing conditions, where high-frequency vibration can be transmitted directly to the lower extremities.

The HGCZ applicability to Foot Transmitted Vibration (FTV) is significantly limited. ISO 2631–1 explicitly states that it does not cover health effects on standing, reclining, or recumbent subjects due to insufficient knowledge and does not consider vascular or neurological effects. Several literature studies (Hedlund 1989; Thompson et al. 2010; Tingsgard and Rasmussen 1994) evidenced a link between high-frequency FTV and vascular disorders of the toes, suggesting that the upper frequency limit of 80 Hz in ISO 2631–1 is too low to adequately assess the potential for vascular issues in the feet, as toe resonance occurs at frequencies between 90 and 60 Hz (K. Goggins et al. 2016). The HGCZ is inadequate for protecting workers from FTV-induced vascular or neurological disorders and in the absence of specific scientific evidence, the acknowledgement of biomechanical similarities between hand and foot (Marrone et al. 2023) suggests the possible use of hand-arm vibration related standards (ISO 5349:* Mechanical Vibration: Measurement and Evaluation of Human Exposure to Hand Transmitted Vibration*, 2001; *ISO/TR 18570:2017—Mechanical Vibration—Measurement and Evaluation of Human Exposure to Hand Transmitted Vibration—Supplementary Method for Assessing Risk of Vascular Disorders*, 2017.) for the quantification of vascular and/or neurological disorders in lower limbs. Therefore, the use of the HGCZ for the assessment of health effects of FTV should be avoided.

#### Cognitive, physiological and motor effects

Halmai et al. (Halmai et al. 2024) systematically reviewed the literature to assess the strength and direction of evidence regarding the acute after-effects of measured levels of occupational WBV exposure on cognition, visual function, postural stability, and motor control. It was concluded that due to a lack of consistency in the characterization of vibration exposure and the assessment of associated effects on functional performance, current evidence is insufficient to provide definitive guidance for updating occupational health and safety regulations regarding WBV.

Savage et al. (Savage et al. 2016) reviewed the effects of exposure to WBV arising from human transit on measured neuromuscular responses (muscle activation and reflex), physiological responses (metabolic, thermal and cardiopulmonary), blood oxygenation (in the cerebral and lumbar blood flow) and biomechanical aspects of human physical function (spinal stability and balance). These effects were compared to control conditions to ascertain changes in performance of physically demanding occupational roles. They found that dependent and independent variables varied so much that it was not possible to draw conclusions on the effects of WBV in the context of occupational performance could not be made. To properly address these changes, specific and occupationally relevant performance tests needed to be included in the study design to help produce meaningful results for practitioners and employers alike.

Accordingly, the HGCZ cannot be relied upon to predict or evaluate acute WBV effects on cognition, visual function, postural stability, or motor control.

#### Biomechanical models

The only way to establish vibration exposure limits grounded in biomechanical mechanisms is through the use of models of the biological system. Patterson et al. (Patterson et al. 2021) reviewed in vivo, ex vivo, and in vitro models for spinal pathologies resulting from WBV; and offered suggestions to address the gaps in translating injury biomechanics data to inform clinical practice. Even at the level of only the spine, the authors concluded that it may take a wider sampling of pathological processes in relevant tissues (muscle, bone, intervertebral disc, and spinal cord) at multiple levels of examination (imaging, physiology, histology, biochemical, gene expression changes) and under different WBV stimulus conditions before the system-level degenerative processes that occur over years of WBV exposure are understood. The authors did suggest that parameters of vibration stimulus (frequency and direction) are highly relevant to specific pathologies, and thus there is a need to systematically evaluate biomechanical effects to establish cause-and-effect relationships.

In this context, ISO 2631–5 represents a significant step toward a more biomechanically grounded approach, as it attempts to relate vibration exposure to spinal loading and the risk of adverse health effects through simplified models of lumbar spine response. However, even this approach remains limited by assumptions regarding posture, exposure conditions, and cumulative damage mechanisms, and is not yet fully supported by comprehensive experimental validation across different populations and occupational scenarios.

### Role of confounding and modifying factors

The response to WBV exposure is not uniform across all individuals. Personal characteristics can modify the dose–response relationship and must be considered when interpreting exposure data relative to established guidance values.

### Posture and vibration transmissibility

Sitting or standing posture as well as the time for which any posture is maintained fundamentally influences the characteristics of transmission of vibration from the machine to and through the human (Magnusson and Pope 1998). In terms of sitting, seated posture leads to inactivity and can be injurious also in the absence of WBV. This relates to disorders of the spine, but also musculoskeletal disorders of the neck, shoulders, arms, hips and knees (Magnusson and Pope 1998). As discussed throughout, WBV can increase the risk of injury from these seated postures, for which the details of the posture and the timeframe are likely to be highly relevant for predicting the effects. Similarly, for standing postures, while less is known on the health effects related to different WBV exposures, the mechanics of the human response is changed when posture is altered (G. Subashi et al. 2006; G. H. M. J. Subashi et al. 2008; Tarabini et al. 2013). The effect of posture is complicated to examine directly due to naturally shifting postures as well as the individual specific nature of posture and comfort (Baker and Mansfield 2010; ISO/TR 10687:2022—Mechanical Vibration—Description and Determination of Seated Postures with Reference to Whole-Body Vibration). ISO 2631–1 explicitly states that the HGCZ is derived from data on seated individuals. While the standard includes guidance for standing postures, it acknowledges that health risk assessments for standing subjects require further validation. There is no published literature supporting the applicability of the HGCZ to musculoskeletal disorders in standing individuals and posture plays a critical role in vibration transmissibility. When the knees are bent, transmissibility decreases (K. A. Goggins et al. 2019b, 2019a), potentially leading to an overestimation of risk if the HGCZ is applied directly.

### Age and exposure history

Several epidemiological investigations involving professional drivers and operators exposed to WBV have identified that age and/or cumulative vibration exposure are associated with the prevalence and severity of LBP outcomes (Bovenzi et al. 2006; Pickard et al. 2022). Age often relates with cumulative lifetime exposure to WBV and other occupational stressors, as well as with the natural progression of age-related degenerative changes in the spine. Furthermore, age is a contradictory risk factor for musculoskeletal disorders in drivers, since some studies find younger drivers (< 40) at higher risk (bus drivers), while others find older drivers (> 45) at greater risk. This discrepancy suggests a potential trade-off between the benefits of driving experience and the cumulative impact of occupational exposure (Pickard et al. 2022).

#### Body mass index and anthropometry

The association between higher Body Mass Index (BMI) and an increased risk of MSD among occupational drivers has also been documented (Pickard et al. 2022). However, as higher BMI is a general risk factor for conditions like LBP, and since both BMI and prolonged sitting are linked to LBP across various professions, it is challenging to definitively attribute this increased risk solely to occupational driving.

#### Lifestyle factors

Smoking is another individual lifestyle factor frequently considered and adjusted for as a potential confounder in studies investigating the health effects of WBV exposure, particularly concerning LBP and lumbar disc herniation (Bovenzi et al. 2017). It is biologically plausible that combining the systemic effects of smoking (impaired disc tissue nutrition and repair) with the localized mechanical stresses associated with WBV could lead to a synergistic effect, accelerating degenerative processes more rapidly than either factor would in isolation.

#### Sex and gender differences

The difference between sex in the reporting or susceptibility to musculoskeletal pain in the context of WBV exposure emerged from different studies. A survey conducted among bus drivers found that female drivers reported consistently higher rates of musculoskeletal pain across all body areas compared to their male counterparts (Steele, 2018). Similarly, a study involving mine workers noted differences in the prevalence of reported musculoskeletal symptoms between sexes (Burström et al. 2017). Conversely, a large cross-sectional study investigating the association between occupational WBV exposure and neck pain in the Swedish general population showed significant associations between frequent exposure to WBV and neck pain among men but not women (Stjernbrandt et al. 2024). Additionally, population data noted a higher burden of neck pain and osteoporosis in women globally. These observations collectively suggest that sex and gender may act as a confounding factor in WBV risk assessment. The reasons for these observed differences are not clear, but may include physiological differences in musculoskeletal structure or pain processing, differences in specific job tasks or exposure patterns even within the same job title. Furthermore, female specific factors such as risks to pregnancy and birth outcomes (Skröder et al. 2021), and the effects of menopause which speeds up bone loss and increases the risk of osteoporosis, require specific investigation. The potential for sex and/or gender-specific responses implies that applying uniform HGCZ levels across mixed- workforces might appear simplistic. If genuine differences in susceptibility or reporting exist, thresholds derived primarily from studies on male-dominated occupations (common in heavy vehicle operation) could misrepresent the risk for female workers. The HGCZ remains a valid first-pass tool for female workers, because the zones purposefully include generous margins to cover individual variability in biological response across the whole workforce. The sex or gender differences reported in the literature do not invalidate the HGCZ, but suggest that the use of the lower boundary of the caution zone as the action level for women in high-risk roles may be reasonable.

#### Interaction effects and multifactorial nature

The multifactorial nature of risk factors associated with work-related MSD is well established (Joseph et al. 2023): among professional drivers there is strong evidence supporting the causal relationship between WBV, awkward posture and lifting tasks (physical risk factors), as well as job stress, job demand and previous pain episodes (psychosocial risk factors).

The reported literature show that WBV is a part of a broader multifactorial system in which mechanical loading, posture, physical workload, and individual susceptibility interact in a non-additive manner. The combined effect of WBV and prolonged sitting or awkward posture amplifies spinal loading beyond what would be expected from each factor alone, while psychosocial stressors may influence pain perception and reporting, further complicating the interpretation of exposure–response relationships. Factors such as BMI or smoking modify tissue tolerance and recovery capacity, thereby altering the individual response to a given vibration exposure. These interaction effects make it inherently difficult to attribute causality to WBV alone and help explain the variability observed across epidemiological studies.

Consequently, risk assessment approaches based solely on vibration magnitude and duration oversimplify the degenerative mechanisms, reinforcing the need for models that explicitly account for confounding and interaction terms as detailed in Sect. ‘‘Limitations of our study’’.

### Applicability of HGCZ across exposure context

#### Land-based vehicles

The A(8) and VDV have been primarily used to predict WBV health risks in drivers of land-based vehicles. However, applying these metrics to other work or leisure situations, or to different types of health conditions, remains questionable (Tarabini et al. 2015). This is particularly important when considering other vehicle types such as watercraft and aircraft, where the characteristics of vibration exposure and the associated health risks may differ significantly from those encountered in land-based driving.

#### Watercrafts and high-speed boats

Ullman et al., (Ullman et al. 2023) summarized the available knowledge on workplace-related injuries and chronic musculoskeletal pain among high-speed boat operators. With only 5 eligible papers, the sources were limited. However, they found common evidence that high-speed boat operators have higher rates of injuries and a higher prevalence of chronic pain than other naval service operators and the general workforce. These findings are likely due to exposure to repeated shocks (Ullman et al. 2022). They reference a prospective longitudinal study (Ullman et al. 2025) to measure the human impact exposure and correlate it with the occurrence and development of pain, which is an indicator of injury.

#### Aircrafts

Systematically reviewing literature related to vibration and the effects on humans in fixed-wing aircraft, Mansfield and Aggarwal (Mansfield and Aggarwal 2022) demonstrated a lack of consistency in vibration measurement method and analysis, limiting comparison between studies. Measurements indicated the presence of vibration components at frequencies attenuated by the ISO frequency weighting filters, which have been identified as relevant for human vibration perception. They recommend standardised reporting of WBV measurements to support the development of databases, including reporting unweighted and weighted data, as well as the effects of human physical and cognitive performance.

Rotary-wing aircraft typically generate lower frequency vibration components as compared to fixed-wing aircraft. A substantial peak is typically observed below 20 Hz for most rotary-wing military aircrafts. These components are still attenuated by the ISO frequency weighting filters. As reported by Gaydos (2012), “low back pain among helicopter pilots has been reported for almost 50 years” with the prevalence ranging from 50 to 92% as concluded in several survey studies. Studies have even shown a prevalence of specific injuries among rotary-wing aircrew such as lumbar disc herniation (K T Mason et al. 1996; Knox et al. 2018). Posture and whole-body vibration have been strongly suggested as causal factors for LBP in rotary-wing aircrew. A few studies have been conducted to quantify aircrew vibration and to assess health risk in accordance with ISO 2631–1. Kåsin et al. (2011) collected pilot seat interface triaxial accelerations aboard six helicopters during 15 separate flight manoeuvres. The A(8) ranged from 0.32 – 0.51 m/s^2^ rms. Regardless, there was a high incidence of reported LBP among these aviators. The authors suggested that posture, in combination with WBV, may be contributing to LBP. The U.S. Air Force Research Laboratory (AFRL) and the U.S. Defense Centers for Public Health—Aberdeen (DCPH-A) collaborated on a project to characterize the vibration exposure and assess aircrew health risk aboard U.S. military rotary-wing/tilt-rotor aircraft. The studies were conducted between 2013 and 2022 on six helicopters and one tilt-rotor aircraft and included multiple flight maneuvers defined by the aircrew as well as multiple data records for most maneuvers. The findings are summarized in (SD Smith 2025). Since the distribution of exposure duration among the flight conditions were not defined, and would vary depending on the mission, the vector sum of the frequency-weighted r.m.s. accelerations during level flight were used to gauge health risk in accordance with the ISO HGCZ. Twenty-nine to 44 short-duration level fight exposure records were collected on each aircraft at each location. Among the level flight exposure records, at least one location aboard each aircraft showed that 75% or greater reached the lower HGCZ boundary in eight hours or less. Five of the aircraft showed at least one location where 50% or greater of the records reached the lower HGCZ boundary in less than 4 h. While the vector sum may have contributed to higher exposure values compared to the study by Kåsin et al. (2011), the type of aircraft may have played a significant role. Chen et al. (Chen et al. 2015) also measure relatively high vibration levels aboard the Canadien CH-147F that entered the ISO HGCZ in less than 4 h.

## Summary and cross-context limitations

The existing literature demonstrates that there is evidence of health risks as a result of occupational exposures to WBV in water and air vehicles. However, there is currently not the strength of evidence to conclude which specific characteristics of vibration exposure signals correlate with health risks, and exactly what the nature of these health risks are (Table 1).

**Table 1:**
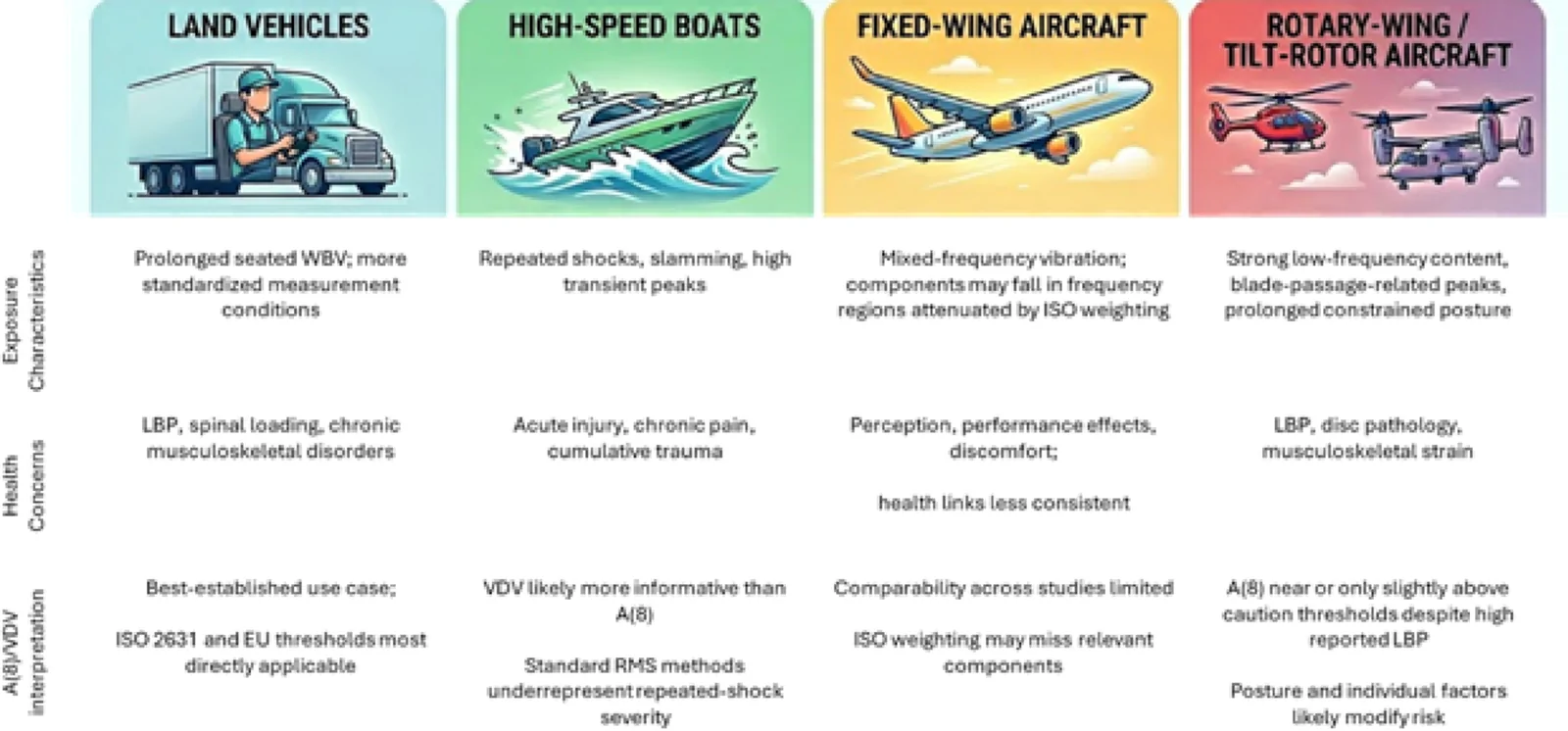
Vehicle classes, dominant WBV exposure characteristics, and likely limitations of ISO A(8)/VDV interpretation

As already mentioned for the confounding factors, the HGCZ boundaries may need to be parametrized for other vehicles or even computed starting from metrics different from A(8) or VDV in order to account for specific vibration characteristics.

## Research recommendations

### Multi metric approach

Current WBV metrics primarily quantify the external vibration environment as measured at the driving point between the occupant and vehicle interface. However, the energy received by specific biological tissues or organs is modified by the body's biodynamic characteristics (vibration transmissibility) and its active physiological responses (muscle tuning and postural adjustments). It is this internal dose and the resulting tissue response that ultimately lead to micro-damage, physiological disruption, and the potential development of adverse health outcomes (Bovenzi et al. 2015). The current vibration metrics are poor proxies for the internal dose and it is therefore necessary to move beyond a single, generalized HGCZ to a more complex system that acknowledges the unique risks associated with different vibration and posture scenarios. Unfortunately, studies have been limited in defining the mechanisms by which whole-body vibration exposure causes specific physical, physiological, and psychological changes at various hierarchical levels of the human body. This knowledge is critical for improving measurement and assessment methods, as well as for the development of predictive models that define the contribution of vibration to specific health risks. This is not an easy undertaking.

Presently, A(8) and VDV are used to assess the risk of LBP in occupants and operators of vehicles or heavy equipment exposed to WBV. Although these metrics have proven useful within a narrow context, the use of a combination of vibration features (including shock indicators, the spectral content distribution of the exposure, the directional components, histograms of acceleration, daily vibration variability, cumulative vibration exposure and other parameters) could provide more comprehensive information on the vibration environment. Machine-learning could also map acceleration patterns to injury risk using biodynamic simulations.

### Biomechanical modelling

The integration of internal load estimators (finite‐element or multibody spine models) into exposure assessment has proven to be more effective than the acceleration-based estimators (Schust et al. 2015). Future standards should incorporate models that estimate biological stresses (not only spinal loads, but also vascular and neurological effects from acceleration data). For instance, an exposed worker’s vibration history should be fed to a spine FE model or to a tissue model to compute cumulative stress indexes, rather than or alongside A(8) and VDV (Schust et al. 2015).

### Frequency weighting

Frequency weightings currently specified in the ISO 2631–1 are suitable for the quantification of low-back MSD of drivers. Different frequency weightings must be proposed for the quantification of the feet neurological and vascular risks. Specific frequency weighting curves must be developed for assessing the risk for different body regions (low back, neck and shoulders, upper limbs, lower limbs) for different postures (sitting, standing). These weightings should ideally be informed by physiological and biomechanical models that predict the transmission and potential damaging effects of vibration across different frequencies on specific tissues and organs and should be derived from biomechanical and biological models. As a further evolution, instead of fixed weighting curves, parametrized curves could be used to penalize frequencies that in a specific posture or with a specific subject have adverse effects on a specific health aspect.

### Probabilistic HGCZ definition

Another essential step forward is transitioning from the current definition of the HGCZ to probabilistic models that estimate the likelihood of developing specific health disorders within each occupational setting. Most existing epidemiological studies focus on reporting the occurrence of a given disorder within selected worker categories, along with the associated exposure conditions. As an example, we analysed literature data (Bovenzi et al. 2006) which included 1391 observations performed on 598 professional drivers of (1) earth moving machines, (2) fork-lift trucks, (3) cranes, (4) trucks and (5) buses. This example is intended to illustrate the proposed methodological approach and its potential for developing occupation-specific probabilistic risk models, rather than to focus on the numerical results obtained from this dataset. Our analyses focused on:

- The LBP intensity, expressed by the Numerical Rating Scale (NRS), a self-reported ordinal measure ranging from 0 (no pain) to 10 (worst imaginable pain) (K T Mason et al. 1996);
- The maximum VDV along the three coordinated axes ($VDV_{max}$);
- The total (cumulative) time in which the worker has been exposed to whole-body vibration.

Descriptive statistics of the above listed variables (mean and standard deviation of the 1391 observations) are summarized in Table 2.

**Table 2:** Descriptive statistics of data used for the analyses reported in the present chapter (Bovenzi et al. 2006)

	LBP intensity (NRS)		VDV_max_ [m/s^1.75^]		Total exposure time [1000 h]	
Driver group	Mean	StDev	Mean	StDev	Mean	StDev
1	3.37	3.35	10.19	3.34	17.5	14.3
2	3.63	3.44	10.27	3.83	16.0	12.8
3	2.47	3.01	8.29	2.95	13.0	11.0
4	3.20	3.41	5.66	0.89	11.0	7.6
5	3.38	3.00	5.86	0.30	21.4	13.8

**Fig. 2:**
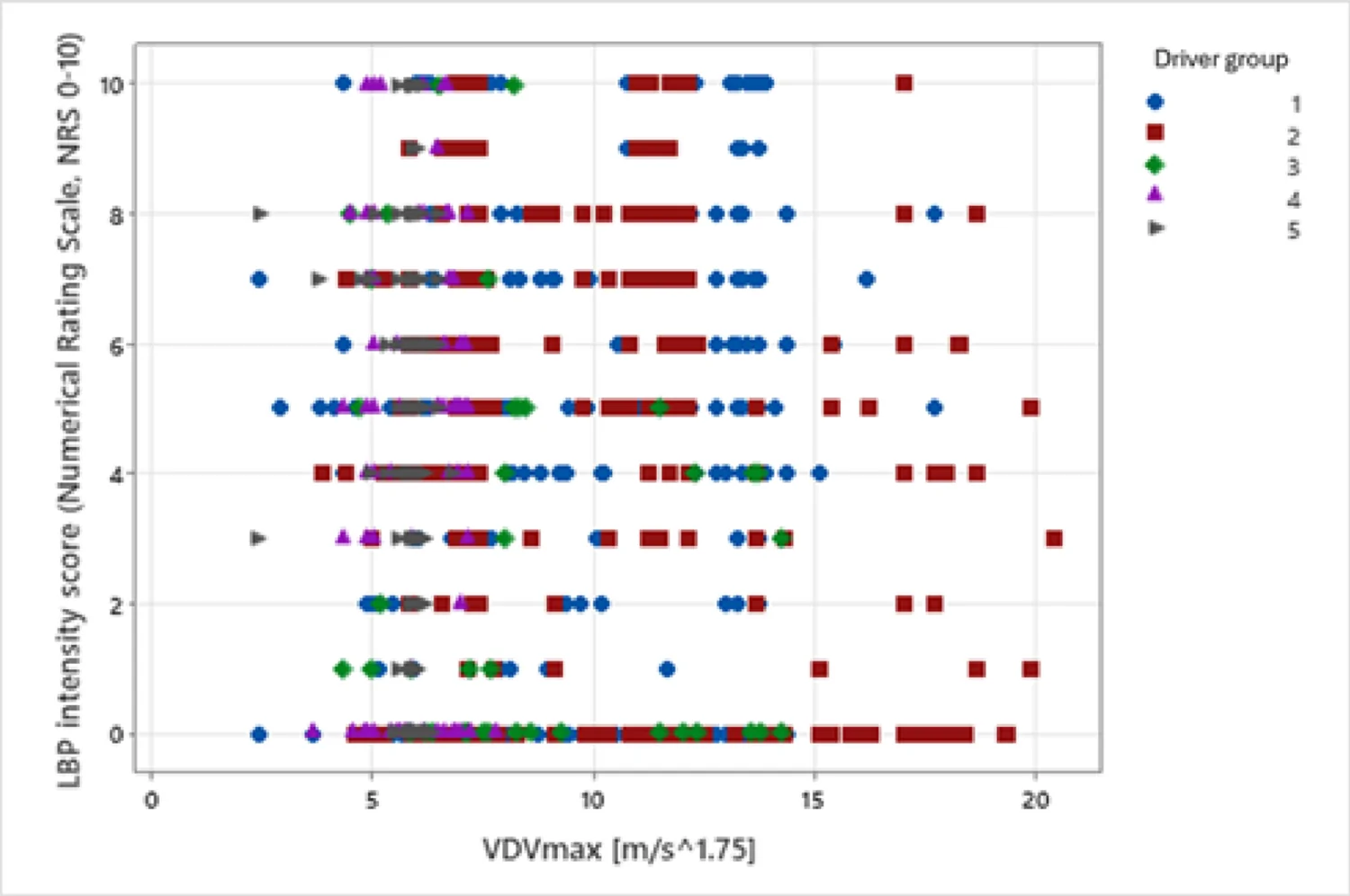
LBP intensity score (NRS 0–10) plotted versus daily WBV exposure expressed as VDV_max_ (highest axis) among drivers of five machinery categories reported by Bovenzi et al., (2006). Each point represents a single worker, characterized by its exposure and the LBP intensity that he experienced

Individual data are shown in Fig. 2.

To explore the relationship between WBV exposure and LBP within specific occupational groups, individual workers can be represented in a two-dimensional exposure space. The x-axis indicates the duration of WBV exposure, while the y-axis represents vibration magnitude, quantified using A(8), VDV, or another appropriate exposure metric. Each point therefore represents one worker, characterized by a specific combination of exposure duration and vibration magnitude, and is associated with that worker’s LBP outcome.

**Fig. 3:**
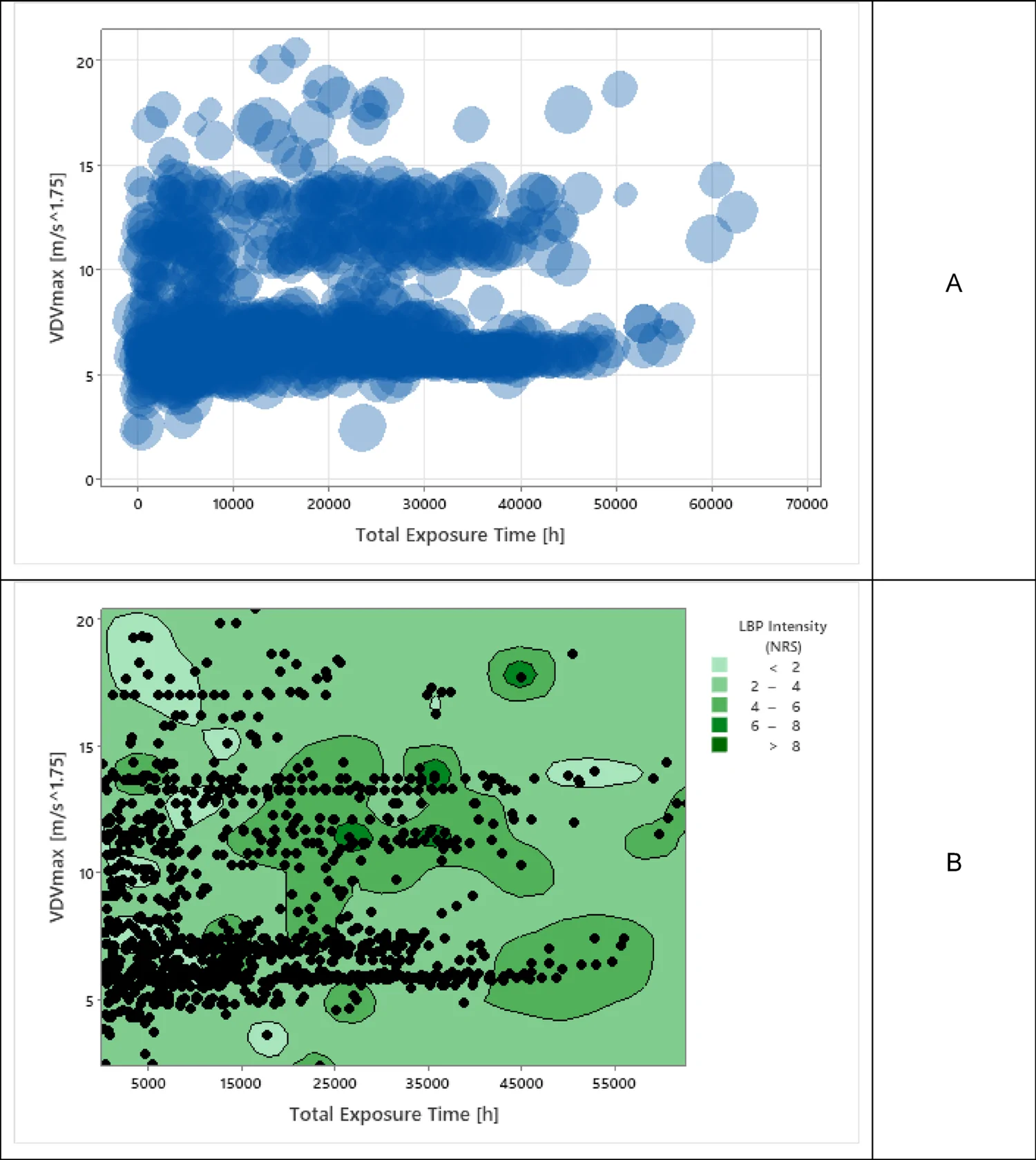
Example of visualization of measured data of vibration exposure (VDV_max_, Total Exposure Time) and health outcome (LBP intensity measured with the Numerical Rating Scale (NRS 0–10) method) using bubble plots **A** or contour plots **B**

In Fig. 3, subplots A and B report, for the workers of Fig. 2, one point for each worker. In subplot A the bubble size is proportional to the NRS: smaller bubbles indicate lower perceived pain, while bigger bubbles indicate high LBP intensity score. In subplot B, the same information is shown as a contour plot, that is a 2D topographical map visualizing the relationship between the response variable (in our case NRS) and the two continuous input variables total exposure time and VDV. The color-shaded regions show the response surface. Unlike the bubble plot, raw data points are fitted by a regression model that can be used to derive the average dose–response relationships. In subplot B of Fig. 3 data points are not evenly spaced, and the software used for the analyses (Minitab 22.5) generates a regular grid and applies linear interpolation across the triangular elements of that mesh to estimate missing response values.

Regression models can be used to expresses the NRS as a function of the independent variables exposure magnitude (VDV_max_) and duration (total exposure time). The interpolating function is:

$$ NRS = \alpha + \beta T - \gamma \cdot VDV_{max} + \delta T \cdot VDV_{max} $$

where *T* is the total exposure time expressed in hours, and ${\mathrm{VDV}}_{{{\mathrm{max}}}}$ is the maximum axis vibration dose value expressed in ${\mathrm{m}}/{\mathrm{s}}^{1.75}$. $\alpha , \beta , \gamma {\mathrm{and}} \delta$ are the coefficients obtained by minimizing the residuals in a least square sense. The regression model, computed on all the 1391 observations, is:

$$ NRS = 3.508 + 1.40 \cdot 10^{ - 5} T - 0.0830 \cdot VDVmax + 2.28 \cdot 10^{ - 6} T \cdot VDVmax $$

**Fig. 4:**
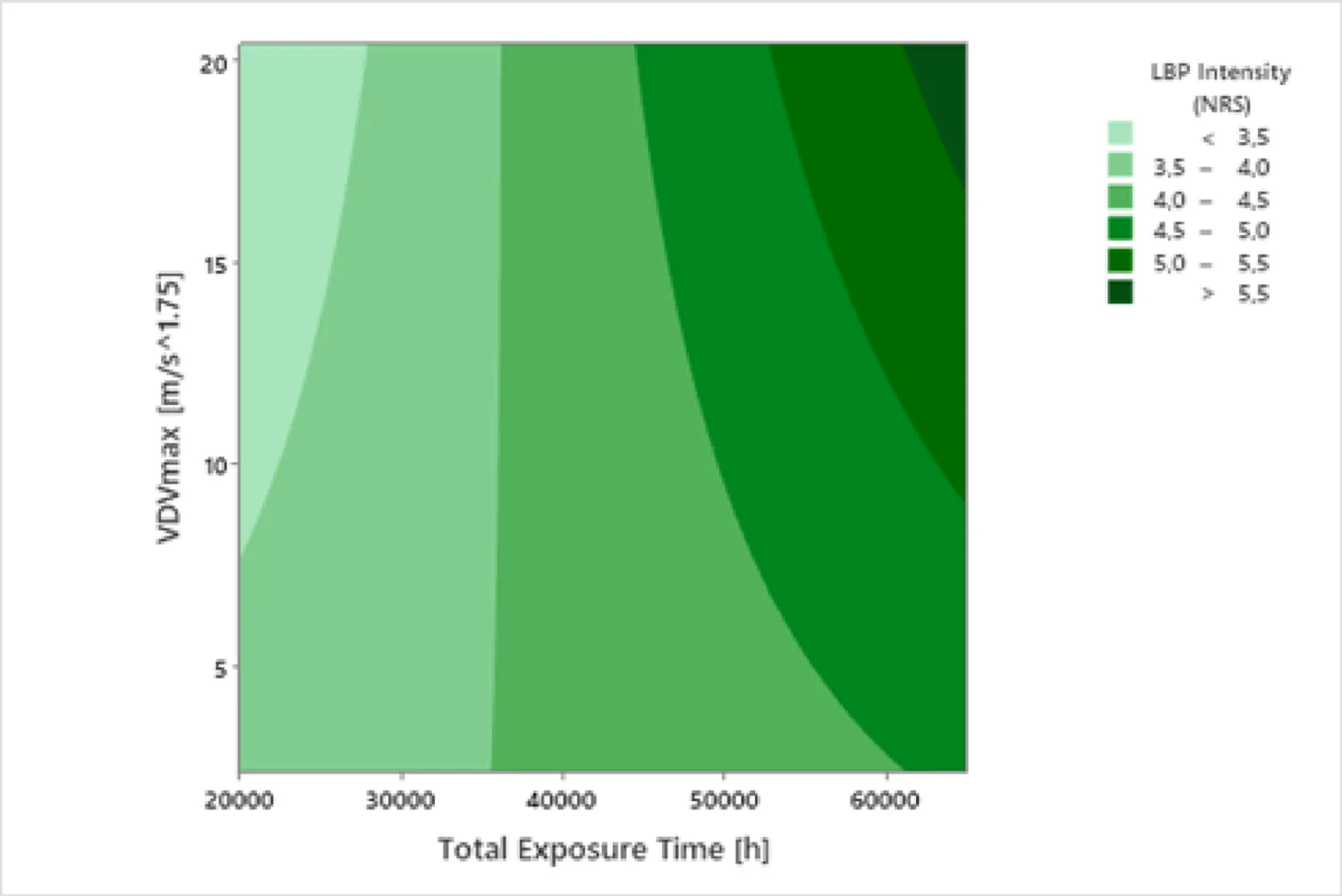
Expected health outcome (LBP intensity measured with the NRS (0–10) method)) deriving from a specific exposure (fitted regression model considering VDV_max_, Total Exposure Time, and their combined effect)

While the individual coefficients should be interpreted with caution, the model (whose prediction is shown in Fig. 4) illustrates how WBV risk may emerge from the combined effect of how strong the vibration is and how long it is experienced, rather than from either factor alone.

These models and the proposed visualizations offer a direct overview into a specific health risk associated with given exposure conditions. Moreover, by adjusting the levels of confounding variables within the model, it is possible to generate scenario-specific risk assessments. To support this approach, it is crucial not only to standardize the reporting of exposure and outcome data (as initiated in ISO 2631–1), but also to establish structured and publicly accessible databases for epidemiological and exposure data.

### Personalized monitoring

Current health surveillance of WBV exposed workers is often limited to either infrequent or periodical clinical visits or questionnaires according to the provisions of the national legislations. To create robust dose–response relationships, a more continuous and objective quantification of the health *response* is needed. Future efforts should explore the development and application of *personalized, non-invasive monitoring devices* capable of quantifying functional impairment or physiological changes relevant to vibration-induced disorders on a daily or even continuous basis. Examples could include devices measuring spinal mobility, peripheral nerve function, or localized blood circulation. Such data would provide a much richer understanding of how an individual's health status changes in response to their specific cumulative daily exposure, potentially allowing for health-based triggers that complement or, in the long term, even inform the vibration dose assessment itself.

### Big data

Integrating detailed individual vibration exposure histories (summarized through multi-metric approaches such as A(8), VDV, and additional descriptors of shock and frequency content) with continuous, personalized health monitoring data across large and heterogeneous worker populations is a key requirement for advancing WBV risk assessment. In this context, a big data framework becomes essential to manage and analyse high-dimensional datasets combining exposure characteristics, task descriptors, individual factors, and longitudinal health outcomes.

To date, most studies—including those presented in this work—rely on regression-based approaches to establish exposure–response relationships. While these methods provide an essential starting point for quantifying associations between vibration magnitude, duration, and health outcomes, they are inherently limited in capturing the complex, non-linear, and multi-factorial nature of WBV-related disorders. The relevant literature presented in this paper confirms that health outcomes emerge from interactions between vibration exposure, posture, task dynamics, and individual susceptibility, which are not fully represented in simplified parametric models.

A paradigm shift is therefore required, moving from purely descriptive or regression-based analyses toward the development of predictive models trained on large-scale datasets. Recent advances in statistical learning and artificial intelligence offer relevant tools in this direction. Techniques such as random forests (Zhao et al. 2024), and neural networks (Aguilar et al. 2024) allow the identification of non-linear relationships and interaction effects, while representation learning approaches (Massotti et al. 2024) can extract latent features from complex sensor data. This perspective is supported by recent work in hand-arm vibration domain (Massotti et al. 2024), where wearable sensors and machine learning have been used to classify task conditions and infer operator behavior, even with limited sensor configurations.

Within WBV research, such approaches would enable the transition from estimating average exposure–response trends to predicting the probability of specific health outcomes under given exposure scenarios. This includes the integration of exposure magnitude, duration, posture, and individual characteristics into unified predictive frameworks. However, this transition also requires caution: model performance is strongly dependent on data quality, representativeness, and proper validation, and there is a risk of overfitting or reduced interpretability compared to traditional models. Therefore, regression-based methods should be regarded as a necessary foundation, but not the endpoint, for developing robust, data-driven risk assessment tools. Ultimately, the combination of physics-based metrics, statistical models, and machine learning approaches may provide the most reliable pathway toward next-generation, probabilistic WBV standards.

## Limitations of our study

This study is subject to several limitations that reflect broader challenges within the WBV research field. First, the availability of data is inherently constrained, as many studies report aggregated exposure metrics without providing access to acceleration signals (mandatory to question the validity of the current ISO 2631–1 parameters) and detailed individual-level information, limiting the possibility of re-analysis and cross-study comparison. Second, there is substantial heterogeneity across the literature in terms of study design, exposure assessment methods, populations, and outcome definitions, which complicates the identification of consistent exposure–response relationships. The access to high-quality medical data remains limited and health outcomes are often based on self-reported measures rather than clinically validated diagnoses. Together, these limitations introduce uncertainty in both the interpretation of existing evidence and the development of predictive models, reinforcing the need for standardized data collection protocols and integrated exposure–health databases in future research.

## Conclusions

The HGCZ was established through expert consensus and remains rooted in a specific historical and regulatory context. As such, its applicability is constrained when extended beyond its original intent. In particular, the HGCZ cannot reliably predict neurological or vascular damage, most musculoskeletal disorders other than low back pain, or the effects of posture, vibration direction, and operational environments such as high-speed marine craft or aircraft. Moreover, its generalized, population-based framework does not explicitly account for confounding factors—including age, body mass index, and sex—that are known to modulate individual susceptibility to WBV.

Notwithstanding these limitations, the HGCZ plays a valuable and practical role in occupational vibration assessment. For the typical assessor, it provides an accessible, standardized, and communicable numerical framework for gauging and comparing the relative severity of WBV exposures across tasks, equipment, and work environments. The availability of a single, general reference metric is a key strength and should be preserved, as it enables consistent decision-making, regulatory alignment, and effective workplace communication. When applied as intended, assessment against the HGCZ can raise awareness of potentially hazardous exposure conditions and serve as a defensible basis for prioritizing engineering, equipment, and administrative controls aimed at reducing daily exposure.

However, rather than being removed, this framework should be refined to better reflect the complexity of human response to vibration, incorporating posture and individual susceptibility, as well as moving toward probabilistic interpretations of risk. A paradigm shift is needed toward approaches that integrate biomechanical and biodynamic models capable of estimating internal tissue loads and resultant biological stress. Frameworks such as those exemplified in ISO 2631–5 highlight both the promise and the current challenges of this transition, including limited uptake by practitioners and the need for clearer guidance to prevent misinterpretation of metrics such as VDV for specific exposure durations. For such model-based approaches to be broadly adopted, they must be translated into assessor-ready tools, supported by training, guidance documents, and clear links to actionable risk management decisions.

In this light, the HGCZ should be viewed not as an obsolete construct, but as an interim mechanism—one that can coexist with, and be progressively supplemented by, more physiologically informed assessment methods as the evidence base matures. Ultimately, expressing WBV-related risk in scientifically interpretable terms remains essential—not only for assessors, but for employers who must be convinced that mitigation is both necessary and in their long-term interest.

## Data Availability

Data and Materials are available upon request.
